# Free trade as domestic, economic, and strategic issues: a big data analytics approach

**DOI:** 10.1186/s40537-023-00722-7

**Published:** 2023-04-14

**Authors:** Moch Faisal Karim, Reza Rahutomo, Ida Bagus Kerthyayana Manuaba, Kartika Purwandari, Tirta Nugraha Mursitama, Bens Pardamean

**Affiliations:** 1 Department of International Relations, Bina Nusantara University, Jakarta, Indonesia; 2 Information System Department, School of Information Systems, Bina Nusantara University, Jakarta, 11480 Indonesia; 3 Bioinformatics and Data Science Research Center, Bina Nusantara University, Jakarta, 11480 Indonesia; 4 Computer Science Program, Bina Nusantara University International, Jakarta, Indonesia; 5 Computer Science Department, School of Computing and Creative Arts, Bina Nusantara University, Jakarta, 11480 Indonesia; 6 Computer Science Department, BINUS Graduate Program - Master of Computer Science Program, Bina Nusantara University, Jakarta, 11480 Indonesia

**Keywords:** Big data analytics, Machine learning, International relations, RCEP, Free trade, FTA, Political economy

## Abstract

This article examines the engagement of domestic actors in public conversation surrounding free trade negotiations with a focus on the framing of these negotiations as economic, strategic or domestic issues. To analyse this topic, this article utilises the use of Twitter as a barometer of public sentiment toward the Regional Comprehensive Economic Partnership (RCEP). We employ topic classification and sentiment analysis to understand how RCEP is discussed in 345,015 tweets. Our findings show that the overall sentiment score towards RCEP is neutral. However, we find that when RCEP is discussed as a strategic issue, the sentiment is slightly more negative than when discussed as a domestic or economic issue. This article further suggests that discussion of RCEP is driven by the fear of China’s geopolitical ambitions, domestic protectionist agendas, and impact of RCEP on the domestic economy. This article contributes to the growing use of big data in understanding trade negotiations. Furthermore, it contributes to the study of free trade negotiation by examining how domestic political actors frame free trade negotiations.

## Introduction

Studies of public opinion on free trade negotiation have become a popular in the scholarship of international political economy. Previous research has developed a variety of individual-level explanations of attitudes toward free trade by utilising approaches such as public opinion surveys and randomised survey experiments [1]. A growing strand of literature focuses on the use of social media to understand debates regarding international negotiation and has yielded interesting findings [2, 3]. Social media platforms, particularly Twitter, arguably enable us to understand the opinions of both the general public and elites on free trade negotiation [4, 5], especially as social media is increasingly used by public officials as a technological communicative tool for diplomacy. It can both represent emotions and provoke emotions within the ongoing political discourse [6]. This enables scholars of international relations to utilise social media to provide further insights into how public audiences perceive free trade, especially during negotiation rounds.

To understand how social media can generate insights on a particular international event, studies are increasingly utilising big data analytics to unpack public sentiments. Georgiadou et al. [4] studied how sentiment analysis has the potential to act as a barometer of public sentiment towards international negotiations, potentially even informing government decision-making. Through the study of Brexit, they found that sentiment analysis of tweets provided information on citizens’ preferences for different possible outcomes of Brexit negotiations. By employing sentiment analysis, Nordheim et al. [5] also found that sentiments on Twitter have become more polarised in comparison to traditional media, showing that discussions on Twitter can better reflect overall public sentiment. These studies shed light on the richness of Twitter data in determining the public sentiment compared with traditional media.

This article examines how Twitter data enables us to further unpack public sentiments towards free trade agreements (FTAs) when contextualised as economic, strategic, and domestic issues. On one hand, scholars of international trade have shown that free trade can be seen as a strategic instrument to enhance foreign policy or security agenda rather than for strictly economic gain [7–9]. In fact, regional trade agreements are largely driven by diplomatic and strategic reasons that have little impact on economic welfare [10]. On the other hand, a growing line of research also establishes that domestic political actors may politicise free trade agreements for their own political agendas [11, 12]. However, how domestic actors engage in public conversation regarding trade negotiation differs when negotiations are contextualised as strategic, economic, or domestic issues remains relatively under-studied.

To fill such a gap, this article applies several big data analytics and machine learning approaches—especially for sentiment analysis, topic classification, and network analysis—to understand public conversations about free trade negotiation. When used alongside sentiment analysis, the approaches of topic classification and network analysis provide a more nuanced analysis of Twitter as a barometer for public opinion. Topic classification allows us to assign a set of predefined categories and identify thematic trends, enabling the testing of existing assumptions about the nature of discourse. Such a technique assists us to classify public discussion on Twitter based on the predetermined conceptual framework. Network analysis further allows us to identify the dynamics of conversations on social networks. This, in turn, enables us to determine how different actors contribute to the debate within the broader Twitter community.

This study examines public sentiment toward the Regional Comprehensive Economic Partnership (RCEP), contributing to the broader field of sentiment analysis by building upon existing machine learning research. Specifically, this study complements research on the use of Twitter data for sentiment analysis, which has been explored in studies such as Sujon et al. [13] and Pak and Paroubek [14]. By examining sentiment toward a specific social and economic issue, the present study extends the literature on the usefulness of Twitter data for sentiment analysis.

Moreover, this study complements research on the use of sentiment analysis in the context of trade negotiations. Research by Mehta et al. [15] and Wu et al. [16] has explored public sentiment toward market prediction using sentiment analysis. This study adds to this literature by examining how sentiment toward RCEP differs depending on the context in which it is discussed. The results of this study can inform policymakers and trade negotiators about the public’s attitudes and perceptions toward RCEP and provide insights into potential areas of support or opposition to the agreement. Overall, this study contributes to a better understanding of public sentiment toward RCEP and the use of sentiment analysis in the context of trade negotiations.

To substantiate the big data analytics approach, we use the Regional Comprehensive Economic Partnership (RCEP) case to illustrate the nexus between online public discussion and FTAs. As the most significant FTA ever negotiated outside of the World Trade Organization (WTO), surprisingly few attempts have been made to unpack the overall public sentiment towards RCEP, with most studies situated within the discussion on the economic, geopolitical and domestic motivations underpinning its success [17–19].

The period of analysis for this study began in 2014, when RCEP was initially announced, and ended in December 2020, almost 1 month after negotiations concluded. We paid particular attention to Twitter discussion on RCEP between 2017 and 2020, due to intensity of debate during this period. This timeline enables us to understand the discussion over RCEP in the initial and mature negotiation phases.

Our study finds that the overall debate over RCEP was neutral. However, when the agreement was framed as a strategic issue, the sentiment was slightly more negative than when framed as a domestic or economic issue though not significant. This article also finds that conversations about trade negotiation as a domestic issue are influenced by politicians and civil society. In contrast, trade negotiation as a strategic issue is dominated by politicians and think tanks, and free trade as an economic issue is influenced by thinktanks followed by politicians. This article further suggests that conversations on RCEP were driven by the fear of China’s geopolitical ambitions, domestic protectionist agendas, and the potential negative impact of RCEP on domestic economies.

This research contributes to two strands of literature. First, it enhances literature on public sentiment on trade negotiation by providing insights through a comprehensive application of big data analytics. Second, it contributes to the study of free trade negotiation by examining public discussions on trade negotiation depending on how it is framed (as an economic, strategic, or domestic issue) by domestic political actors.

This article is structured as follows. The following section elaborates on the current debate on public opinion and free trade and the importance of incorporating big data analysis from social media. The third section provides context to understanding RCEP as a strategic, economic, and domestic issue. The fourth section provides the methods of analysis for our application of the machine learning approach. The fifth section examines the application of machine learning to understanding public sentiment.

## Literature review: public sentiment and trade negotiation

International relations scholars have long investigated the relationship between public opinion and free trade. Most studies have been primarily framed within the discussion of the extent to which free trade may provide economic gain. This stems from the assumption that FTAs are important policy tools for economic development. The logic is that FTAs increase a country’s degree of trade openness, boosting economic growth due to trade promotions. Proponents of trade liberalisation argue that this is likely to provide economic gain because considerable tariff reductions on certain sectors may benefit net importers of products.

The consensus of political economy literature has suggested that individual trade preferences are mainly driven by the logic of trade liberalisation. This means that individuals who see their country as having comparatively advantaged sectors that will gain from trade liberalisation tend to favour such agendas, and vice versa. Support for free trade is not only driven by material economic gain but also perceptions of how a country’s economy as a whole is affected by trade [20]. As a result, much literature has focused on the labour market (Heckscher–Ohlin model), exploring how high-skilled workers are more likely to support free trade but low-skilled workers are less likely to support such endeavours [21] as well as how opinion vary between different types of occupation [22]. Other literature analyse the types of industries present, finding that where domestic industries have a strong orientation toward exports, countries are more supportive of free trade (Ricardo–Viner model) [23].

Another strand of literature pays more attention to non-economic factors that drive preferences toward free trade. In his study regarding the effect of economic integration agreements on international trade flows, Kohl [24] found that half of such agreements have had no discernible impact on trade at all. This finding shows that FTAs should not be seen as instruments to facilitate trade liberalisation, gain market access, and stimulate economic integration, but instead have other non-economic objectives. Spilker et al. [25] also found that non-economic considerations, such as environmental and labour standards and labour market access, influence citizens’ preferences and economic factors.

It has been widely argued that free trade has been utilised to enhance countries’ strategic and foreign policy considerations [26, 27]. This is because free trade has been seen as a tool to promote peace by removing protective barriers to international commerce in order to limit aggression in foreign policy [28]. In the case of US activity in the Middle East, for instance, FTAs have been mobilised as an instrument for foreign policy instead of in pursuit of economic interest [29]. Capling [8] has shown that the proliferation of FTAs in the Asia–Pacific region has been driven by a pragmatic response by local governments to the lack of progress by multilateral trade institutions such as the WTO. Sohn and Koo [30] demonstrated how security and strategic factors, along with economic considerations, influenced the negotiation phase of the Korea–US FTA, showing how FTAs can become instruments to securitise relations between two countries. The impetus for initiating trade agreements is mainly influenced by both countries’ aspirations to re-securitise bilateral economic ties. However, the stalemated ratification process in the US shows that the American domestic audience are not interested in using trade liberalisation as an instrument for the US’ broader strategic interests, even when applied to the country’s allies.

Given the importance of strategic considerations in enacting free trade, international political economy scholars should examine domestic audience opinions on FTAs with regards to strategic and foreign policy factors. By reviewing voting patterns in the US House of Representatives, Milner and Tingley [9] found that foreign policy consideration has more apparent influence on congressional votes relating to trade. Moreover, they also found that liberals and left-leaning constituencies are more favourable to aid rather than trade, while conservative legislators prefer trade over aid. DiGiuseppe and Kleinberg [31] further showed that when security considerations are taken into account, the appeal of economic gain may shrink, and that elites might be a constraint in liberalising trade if they perceive an agreement could benefit a perceived adversary.

Scholarship on free trade has also established the importance of domestic politics in affecting public opinion. A protectionist coalition might arise due to elite persuasion and policy campaigns, particularly those using powerful protectionist interests [32]. Other scholarship looks at the importance of ideational factors in affecting how the political elite respond to free trade. On the one hand, scholars have argued how ideas about the free global trading system have become rooted in policy-making discourse [33], underpinning the stability of free trade despite the ongoing resistance. On the other hand, ideological preferences of particular government would have an impact on its trade policy position [34].

To understand the link between public opinion, domestic politics, and free trade, some scholars emphasise the importance of elite cueing. Hicks et al. [35] show that due to their organisational power, political parties can effectively mobilise voters and frame their message. Arguably, citizens rely on cues provided by political elites, especially political parties, when forming their views on trade agreements. The literature on cueing in political communication research identifies two conditions for elite or party cueing work: lack of information, and trust. Citizens that are knowledgeable about a trade agreement do not need to take cues provided by political elites, while other citizens tend to follow only the cues provided by the political party they trust. The findings offer some support for the existence of cueing effects that are conditional on information and trust [36].

Given the above discussion, scholarship in the international political economy has established how free trade can be an economic, strategic, and domestic issue. Druckman et al. [37] argued the way in which a particular topic is presented to the audience influences how the audience might react. The framing effect occurs when speakers focus on specific relevant considerations that cause individuals to focus on those messages. The way in which free trade is framed then matters. While most studies focus on public opinion as a method to understand such framing, scholarship has increasingly turned to social media to capture public perception.

With the continually growing use of social media, many scholars see the global social media sphere as a transnational public sphere for socio-political discourse [38–40]. As suggested by Casero-Ripollés [41], the conversation on social media includes journalists, politicians, and more decentralised actors, enabling scholars to better understand the production of political information and public opinion. In fact, social media can provide more nuanced insights on public opinion than other areas of conversation, because it can capture any polarisation that occurs in the social perception toward a particular issue [42].

Social media is important for understanding international issues such as free trade. Ashbrook and Zalba [43] argued that social media has three important roles in international negotiations: (1) as an additional communication channel, (2) as an information source for diplomatic reporting where media access is scarce; and (3) as a diagnostic tool to explore both diplomatic reporting and the impact of negotiations. Duncombe [44] showed the transformative impact of social media in international negotiations during the 2015 US–Iran nuclear deal. Duncombe showed how the use of Twitter by Iranian officials helped the Iranian government reveal their desire for a positive outcome.

Recent literature has focused on the importance of utilising the machine learning approach to unpack the wealth of social media data in understanding on-going trade negotiations. The study by del Gobbo et al. [2] utilised Twitter data in order to uncover the topics that attracted the most attention from users during Brexit negotiations. Georgiadou et al. [4] showed that Twitter can be used as a real-time barometer of public sentiment towards international negotiating outcomes. Using the case of Transatlantic Trade and Investment Partnership, Maireder et al. [45] found that Twitter can also be used to understand the patterns of how online actors diffuse political information through social networks.

The use of social media can also provide avenue to further deepen literature on the political economy of trade policy especially in analyzing actors involved in the negotiations. The literature has established that domestic interest groups and political institutions play a crucial role in shaping trade policy [9, 46, 47]. Others argue that international factors, such as the power of multinational corporations and the rules of the international trading system, have a greater influence. Domestic actors can affect trade policy outcomes in various ways, depending on their interests, resources, and strategies. Studies have suggested that domestic actors can mobilises their resources to support or resist particular trade policy. Interest groups, such as business associations, labor unions, and consumer groups, can lobby policymakers to adopt or oppose certain trade policies [48]. There is an increasing tendency where domestic actors can use the media to shape public opinion and influence policymakers [49]. For example, interest groups can use social media or advertising to promote their positions on particular policy issues [50]. This article enhance the literature by showing how big data analytics can provide insights on how each domestic political actors frame their interests in social media.

Building upon these studies, in this article we examine social media conversations regarding free trade, specifically when free trade is framed as an economic, strategic, and domestic issue. Understanding this public sentiment allows us to further unpack audience’s feelings towards free trade and identify recurring themes in public opinion when discussing negotiations. Lastly, through social network analysis, we can further understand which actors are more likely to frame trade as an economic, strategic, and domestic issue.

This article aims to synthesise the literature on free trade with the growing machine learning approach. To illustrate its usefulness, we examine public discussion on RCEP. Before applying the machine learning approach, the next section will discuss RCEP as an economic, strategic, and domestic issue.

## Understanding RCEP: economic, strategic, and domestic issues

RCEP is a comprehensive regional economic partnership in Asia, initiated by Indonesia during its leadership of ASEAN in 2011. This cooperation consolidates the five FTAs that already existed between ASEAN and its six trading partners. Negotiations were declared complete on 11 November 2020. Fifteen 15 countries joined: the 10 ASEAN countries (Indonesia, Malaysia, Thailand, Vietnam, Singapore, Myanmar, Brunei, Cambodia, Laos, and the Philippines) and five ASEAN trade partners (China, Japan, South Korea, Australia, and New Zealand).

The primary determinant for the initiation of RCEP was strategic. Key to this is the declining centrality of ASEAN in the region, particularly in the areas of trade and connectivity [19]. In trade, ASEAN’s central position has been overtaken by contemporary developments, ASEAN + 1 FTAs have seen limited usage; and ASEAN’s external partners have negotiated several bilateral FTAs that excluded ASEAN. These FTAs are more ambitious and contain more specific commitments than provided by ASEAN’s economic cooperation agenda, which limits ASEAN’s substantial leadership in external negotiations. Additionally, the ASEAN Economic Community has not made significant progress on crucial issues such as intra-regional trade integration in recent years. Altogether, this has meant that RCEP has resulted in East Asian trade relations that are more multipolar and rely less on ASEAN as a fulcrum and norm provider. The Northeast Asian partners also leveraged RCEP negotiations to further trilateral talks, questioning the substantial ASEAN centrality [51].

Prior to RCEP’s inception, the idea of establishing a broader mega-regional integration project had been proposed by several major powers in East Asia. Japan had once proposed the establishment of the East Asian Community (EAC) through a policy initiative called the Comprehensive Economic Partnership for East Asia (CEPEA). This proposal was not received very well, particularly in China. In fact, China has also proposed its own initiative, the East Asia Free Trade Agreement (EAFTA). However, the initiatives proposed by Japan and China, despite being highly significant for the development of cooperation in East Asia, were met with scepticism by ASEAN countries, particularly Indonesia. This is due to the fear that the initiatives could be a part of a strategy by the major powers in the region to influence the middle and small powers in Southeast Asia. To ensure that the driving seat for East Asia regionalism is still ASEAN, Indonesia raised the idea of creating RCEP when it became the Chair of ASEAN in 2011.

Economic issues were also key to the development of RCEP. ASEAN countries had become aware of the additional economic benefits to be gained from additional opening up. The expected additions were new market access that had not been achieved through the ASEAN + 1 FTAs, additional spill-over effects from the opening of markets between fellow FTA partners, bilateral FTAs between individual RCEP and non-RCEP countries, and improved trade efficiency between member countries (through a cumulative mechanism with the agreement on the Rules of Origin (ROO), RCEP trade facilitation scheme, and other provisions).

Economists argued that RCEP will increase investment to less-skilled and lower-cost countries such as Vietnam, Laos, and Cambodia. Economists predicted that RCEP would provide economic benefit mainly through the inverse relationship between exports and exchange rates, making countries more competitive in international markets [52]. For China and Korea, RCEP might increase the trade of China by 1.5% while Korea will increase to 0.9% [17]. In the long run, lower exchange rates will reduce imports and raise exports to compensate for the increased cost of exports. Hence, economists, in general, believe that the long-term gains for countries joining RCEP outweigh the short-term losses from not joining the RCEP. Vines [18] argues that RCEP might offer the possibility of outward-looking liberalisation. However, it will involve a staged agreement in which it needs to address institutional issues in the first 10 years of the liberalisation process.

In the case of Indonesia, RCEP will lead to a 20% increase in investment as well as an increase in GDP [53]. In addition, 60 million Indonesian MSMEs will be positively affected by this trade cooperation because RCEP makes it easy for them to export their products. Indonesian exporters will only need to use one type of certificate of origin to be able to export to all RCEP member countries. Indonesian policymakers see that Asia has always been the factory, market, and engine of world economic growth. Therefore, Indonesia must take advantage of the RCEP momentum to increase exports, especially as the majority of Indonesia’s exports have always been to RCEP member countries.

However, despite its significant economic potential, RCEP has been clouded by domestic interests. For example, the agreement has been perceived negatively by the domestic Indonesian audience, where there is significant pressure from key ministries (such as the Ministry of Industry) as well as other domestic economic interests to be more protectionist. Despite this, the persistent efforts by the Indonesian foreign policy establishment as well as the Ministry of Trade to initiate the RCEP indicate that the agreement is not merely the product of economic imperatives. As revealed in interviews with high-ranking officials within the Ministry of Trade, despite the economic opportunity rhetoric, since the beginning RCEP has been part of Indonesia’s foreign policy agenda to pursue its geostrategic interests in East Asia. This geostrategic concern was why Indonesia was reluctant to join the now-defunct US-led TPP.

While Indonesia has now ratified RCEP despite domestic concerns, India’s rejection of RCEP is due to domestic pressure. Gupta and Ganguly [54] show that the move was mainly driven by the ideological narrative of the importance of self-reliance. For India, RCEP would negatively impact Prime Minister Narenda Modi’s ‘Make in India’ policy that aims to envision India as a manufacturing powerhouse. Protectionist lobbyists, especially from the aluminium, copper, auto and steel industries, have thus successfully lobbied Modi’s government not to join the RCEP [55]. Fears over RCEP flooding the country with more Chinese imports is part of the general paranoia in India over implications of trade liberalisation agreement and growing anti-China sentiment in the wake of border tension between the two countries. Thus India’s withdrawal from RCEP is aimed to protect its domestic market from flooding imports and no gains in services.

## Methods: a big data analytics approach

The above discussion has established how RCEP can be seen as a domestic, economic, and strategic issue. To explain the process in breaking down the three categories of issues more explicitly, the research likely employed a systematic approach to the topic classification. This approach may have involved using machine learning algorithms, such as natural language processing (NLP) techniques, to identify and categorize the issues discussed in the tweets related to the RCEP. For example, to identify key phrases and words associated with the different categories of issues (i.e., economic, strategic, and domestic). These phrases and words may have been pre-determined based on prior research or established frameworks for categorizing public opinion on the trade agreement.

The algorithm may have also taken into account the context in which these phrases and words appeared, such as the tone and sentiment of the tweet. By analyzing the language used in the tweets, the researcher was able to classify each tweet into one of the three categories of issues. By involving an iterative process of refining the algorithm based on initial results and testing. This approach helps to ensure that the categorization of issues is accurate and consistent.

We will now further unpack how public sentiment toward RCEP varies depending on how the agreement is contextualised. To do so, we utilise a supervised machine learning approach to unpack Twitter discussions on RCEP. Three trained undergraduate students labelled 1000 sample tweets into three categories—economic, strategic or domestic—and to add whether they feel each tweet has a negative or positive sentiment towards RCEP.

We label tweets as economic when the tweets talk more about RCEP in an economic context. Our domestic category is for tweets that discuss the process and domestic implications of RCEP, such as public pressure or interest groups. Meanwhile, we use the strategic label to categorize tweets that talk about interactions between countries, diplomacy between countries, and geopolitical implications. Negative sentiment is used when the content of the tweet is more inclined to reject or be critical of RCEP. At the same time, positive sentiment is determined to exist when the content of the tweet feels RCEP will have a good impact, and negative when they feel it will have a bad impact (Table 1). A problem here is that the sentiment may be judged differently depending on the reader. To resolve this issue, we conducted intercoder reliability tests where we only took labels where all three students were in agreement. We specifically request the students to focus on the meaning behind the statement. Example of tweet number 7 could pass as neutral statement. However, given that India’s response to RCEP is rejection, hence we label this tweet as negative.

**Table 1:** Labelling for topic classification and sentiment

No.	Tweets	Label	Sentiment
1	#RCEP proposals threaten affordable meds from India	Domestic	Negative
2	PM Modi’s decision not to join #RCEP is an admission that even the prospect of joining a massive regional trade agreement isn't incentive enough for New Delhi to launch deep economic reforms, says Mihir S. Sharma: https://t.co/urhKYuGWQr	Domestic	Negative
3	RT FollowCII: in #RCEP, Indian Industry must identify its interests, investments in member countries and prepare well. We can calibrate our liberalization and demand market access ~ Sudhanshu Pandey, Additional Secretary, DoC_GoI at #CIIinteraction	Domestic	Positive
4	RT FollowCII: PMOIndia PiyushGoyal DoC_GoI PIB_India PTI_News DIPPGOI PiyushGoyalOffc A lot of focus has to be on enhancing competitiveness of #India & Indian Industry in the context of #RCEP & other trade agreements that India may need to enter into in the near future. ~ Sa…	Domestic	Positive
5	#IndiaAbroad: Japan on Sunday gave clear indications that efforts were on to make India join the ambitious Regional Comprehensive Economic Partnership (RCEP). #RCEP #Japan #India #NarendraModi @narendramodi https://t.co/naTNU2hCUK	Strategic	Positive
6	#2020: Pres #XiJinping’s visit to Myanmar ushered in a new era for bilateral ties. #RCEP was signed. Premier #LiKeqiang attended the leaders’ meetings for East Asian cooperation. China and #ASEAN marked a historic milestone of becoming each other’s largest trading partners	Strategic	Positive
7	India’s decision on #RCEP reflects its assessment on current global situation and fairness of agreement: MEA READ: https://t.co/v1LoEkqGvDhttps://t.co/v9f8Fjg34d	Strategic	Negative
8	The biggest concern of India with RCEP at this juncture is not merely the economic reasons, but more geopolitical: the existence of China. This is something that other nations in the #RCEP also need to ponder upon, contends Nilanjan Ghosh: https://t.co/1yOI4yfsIW	Strategic	Negative
9	After the signing of the agreement in 2020, the Chinese market will provide more opportunities to RCEP countries. #Globalization #RCEP https://t.co/zDsyFP8Pe7https://t.co/jnjElKGpR3	Economics	Positive
10	#ExpressExplained | trade typically enhances wellbeing across the world by forcing countries to do what they can do most efficiently and procure (import) from the rest of the world what they cannot produce efficiently. So, why did India back out of #RCEP? https://t.co/vQzQ4JYKIN	Economics	Positive
11	#MCopinion | a national consensus is evolving that FTAs affect domestic manufacturing, cause increase in imports, cripple the ‘Make in India’ scheme and have no significant impact on the investment scenario, says Arjun Raghavendra M. #RCEP #FTA @NITIAayog https://t.co/52eSW4OOZE	Economics	Negative
12	Those who advocate joining #RCEP on grounds of integration with global value chain should take note of this trade composition. Global or local, production value chains are not the homogenous string of production process, points out @Abhijit_M007: https://t.co/x8mpTwpYqe	Economics	Negative

Having established our labelled data, the research relied on the utilization of several programs (Twitter API v2, TWARC2, and MongoDB) in data acquisition. Usage of Twitter API v2 can deliver a huge benefit for academic research as the utilisation of the product in the research track allows a user to crawl up to a maximum of 10 million tweets per month. This new API offers more features that enable users to do more with their data. One of the powerful features is a full-archive search endpoint with a volume cap of 10,000,000 tweets per month. The full-archive search endpoint allows users to collect the data of matching public tweets since the first tweet was sent on 26 March 2006. This endpoint allows a maximum of 300 requests per 15 min and a maximum return of 500 search results per request. Hence, using this endpoint can provide a maximum possible return of 14,400,000 tweets per day. Utilizing this new API endpoint allows users to gather public tweets data that matches their search query with at least 10,000,000 search results of tweets, including backdated tweets. Meanwhile, TWARC2 simplifies Twitter data crawling by providing designated commands if the API key is embedded, and to manage data crawling outputs, MongoDB offers a user-friendly approach.

We generated 1,669,621 tweets that contained the term ‘RCEP’. However, almost two-thirds of the tweets (1,149,402) were in Japanese, followed by English 345,286, Hindi 58,770, Korean 28,753, Indonesian 16,190, and Chinese 15,206. Given our model only works for the English language, we only include tweets in English. We further clean the data from non-related tweets. Our final corpus contains 345,015 tweets.

**Fig. 1:**
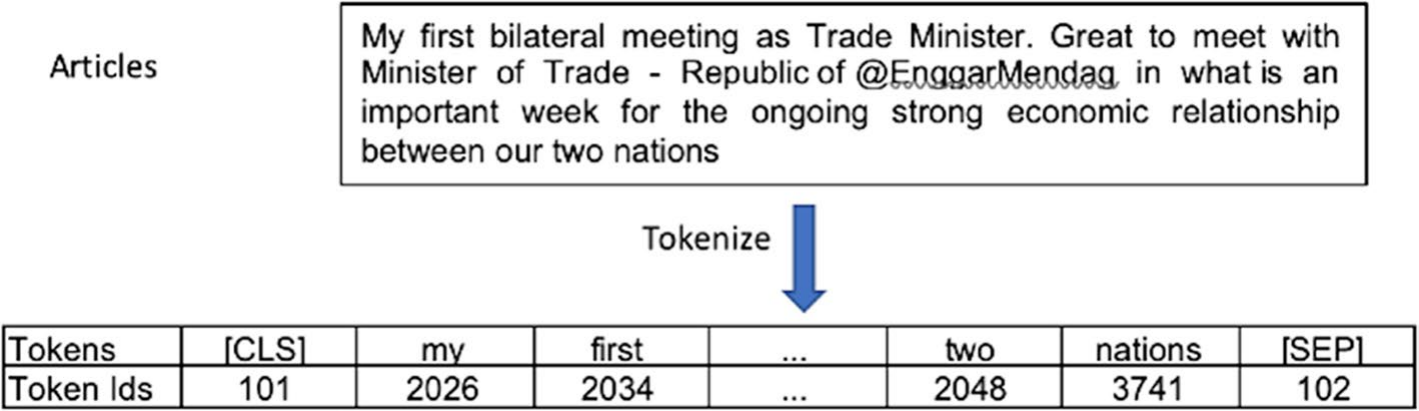
An example of the tokenisation process

We used a pre-trained tokeniser to convert texts into sub-words tokens using the BERT-base-uncased version of BertTokenizer to prevent out-of-vocabulary issues. The tokenising process has been implemented into text classification and sentiment analysis for discussion about RCEP. The [CLS] token is added at the beginning of articles tokens, and the [SEP] token at the end. The token IDs of each token have been converted. Figure 1 illustrates our tokenisation process in our pre-processing phases.

The original texts were fed into the tokeniser as inputs in our study. The case-folding was done instead of uncased models by reducing all letters to lower case. TorchText first creates two fields: a Text field and a Label field. Using the Text column, the tweets article will be loaded, and the Label column will be the target of the tweet. For BERT input, we limit the article to the first 128 tokens. Then we create a TabularDataset by concatenating two fields in the CSV file to generate our training, validation, and test sets.

Given not all Twitter users put their location, we randomly sampled the user to further investigate their location. In describing the distribution of users and the total tweet numbers based on countries, two Python package libraries, *Geopandas* and *Basemap*, are utilised. These two packages can be used to map the user’s location based on geo-location (latitude and longitude) information and produce the heatmap colour to distinguish the distribution of the tweets of each country.

**Fig. 2:**
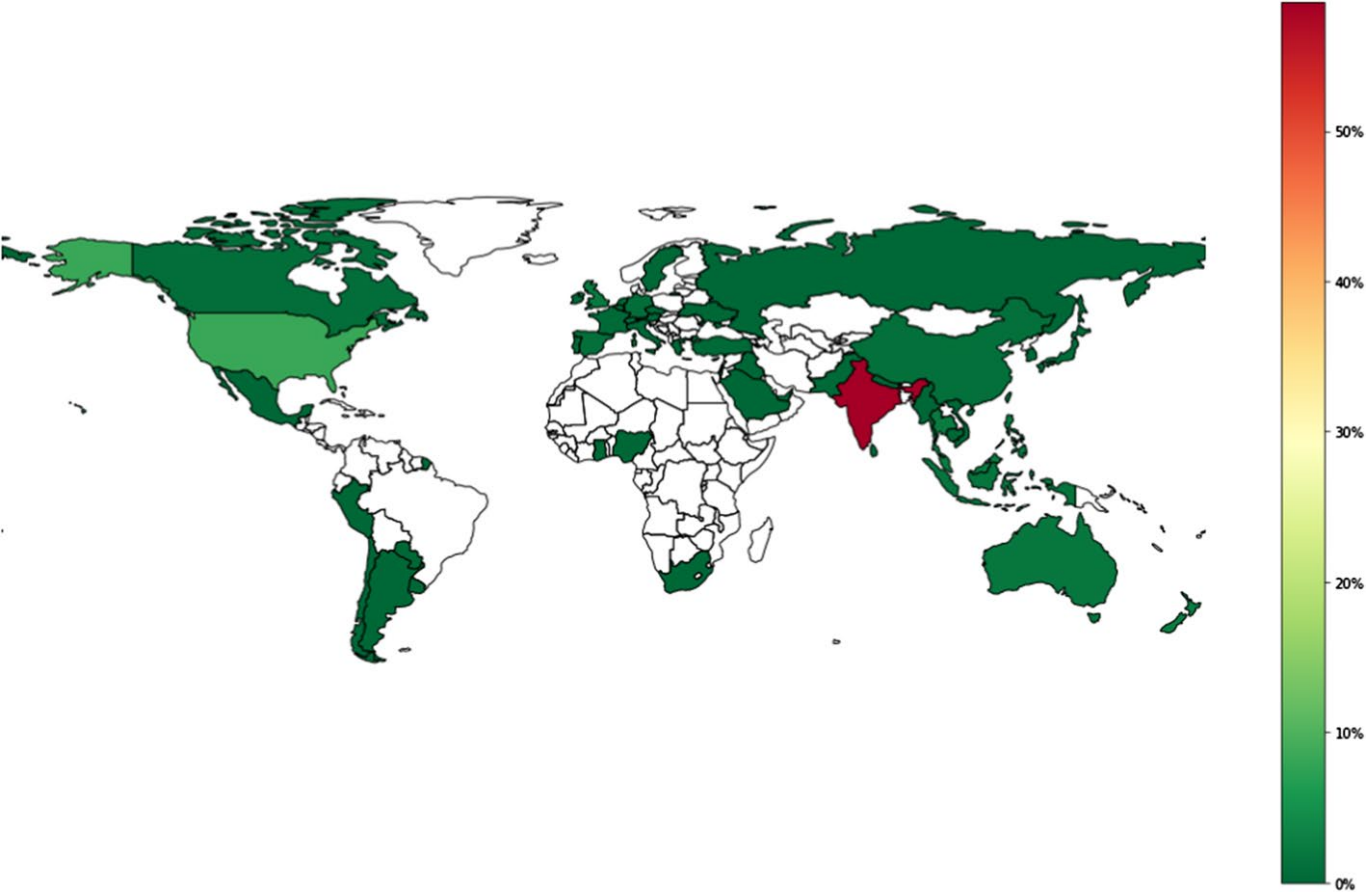
Distribution of Twitter users for #RCEP keyword

We find that most tweets come from India with 58%, the United States 8.5%, Singapore 4.9%, and Malaysia 2.7% (Fig. 2). This distribution is indeed biased toward English speaking countries, especially India. As a result, our findings would be driven by how India’s audience perceived RCEP. The network analysis for such corpus will also be skewed towards India’s public figures.

**Fig. 3:**
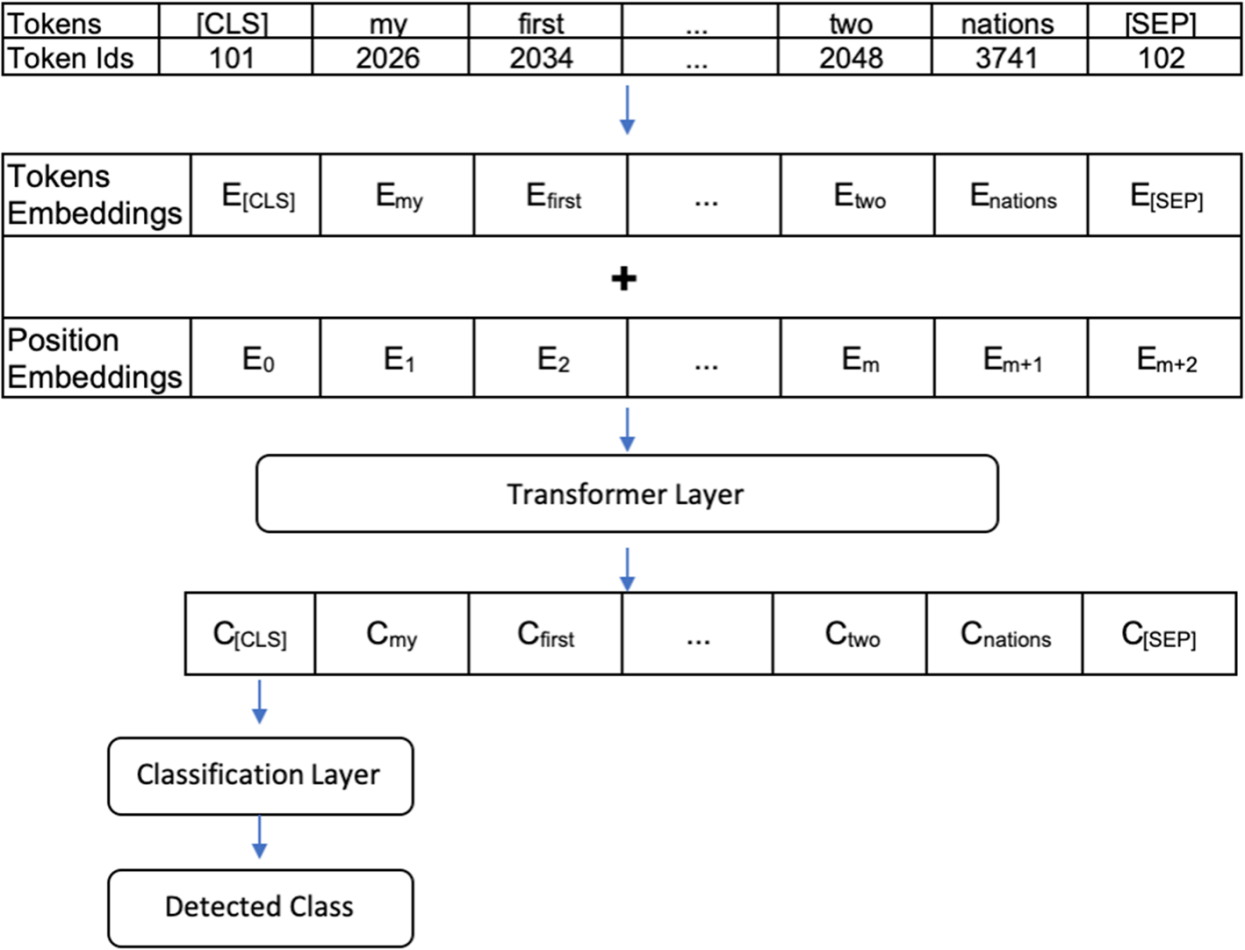
Fine-tuned BERT Architecture for Analysis of RCEP

To conduct topic classification, we utilise Bidirectional Encoder Representations from Transformers, or BERT. BERT is one of Google’s most famous Natural Language Processing (NLP) models for generating state-of-the-art results in a variety of NLP tasks. The BERT model consists of a transformers algorithm that is pretrained on English language data in a self-supervised fashion. We adapt fine-tuned BERT-base-uncased from BERT architecture in to solve the classification task regarding discussions on RCEP. Our proposed fine-tuned architecture is depicted in Fig. 3.

We assigned token embeddings representing the meaning of each token, segment embeddings to discriminate the title and body of the article, and position embeddings covering the token position in our input sequences. The summation of these embeddings was fed to the Transformer layer of BERT. We used the top context [CLS] token as a representation of sequence tokens. Then, we added a classification layer to detect whether an article is a strategic issue, domestic politics issue, or economic issue. We used original BERT models trained on the different corpus.

**Fig. 4:**
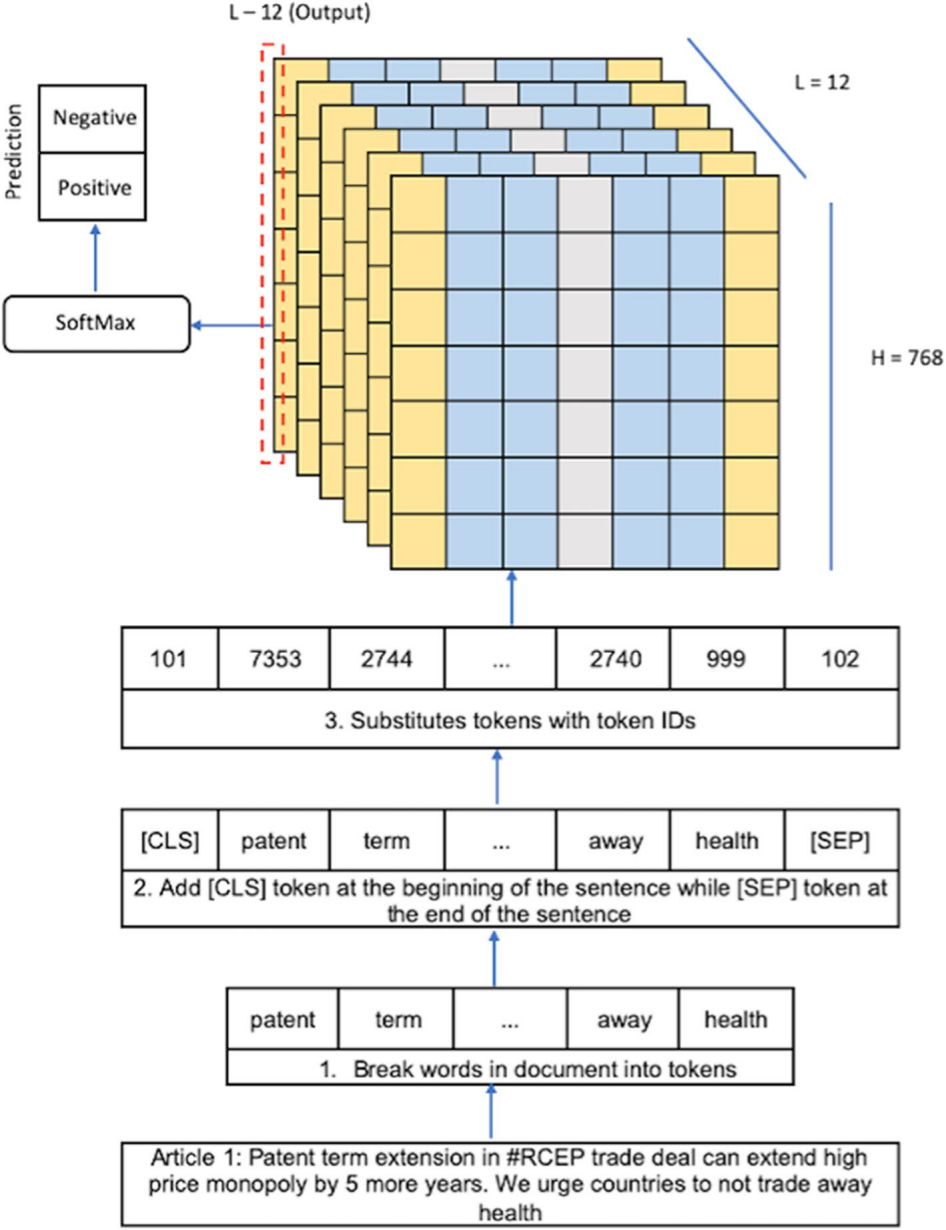
BERT tokenising and fine tuning for sentiment analysis of RCEP

The process of conducting sentiment analysis on the RCEP system can go through the same process as text classification. However, we change the output to sentiment analysis, which is positive or negative. After the tweets are processed using the BERT-based-uncased tokeniser, the pre-trained BERT is used to carry out the sentiment analysis process. Figure 4 shows how the fine-tuning model is used for classification. The accuracy of our topic classification model is about 71%, while our sentiment analysis model is around 85%.

## Findings

### Overview of topic classification of RCEP discussions

**Fig. 5:**
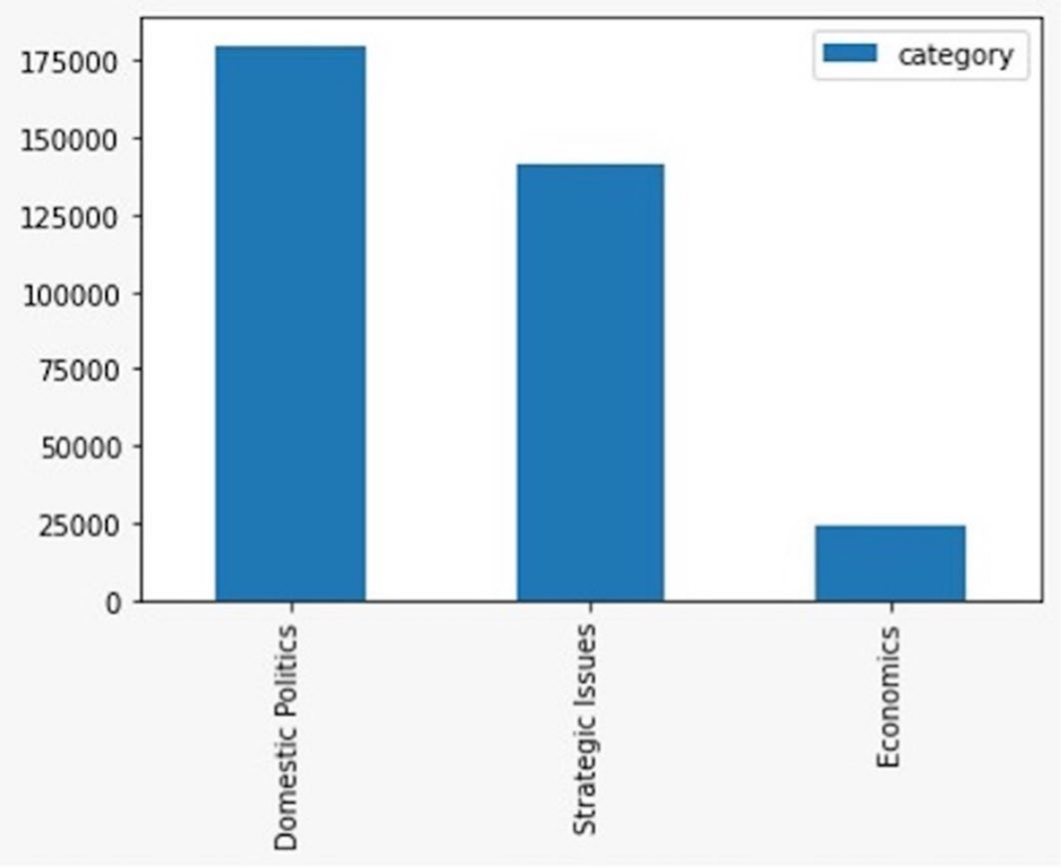
Topic classification of RCEP discussions

**Fig. 6:**
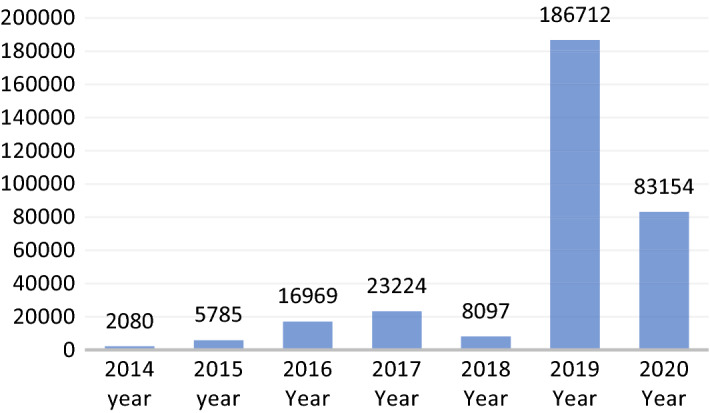
Distribution of RCEP tweets per year

Our machine learning model revealed that discussions of RCEP are dominated by domestic issues, followed by strategic then economic issues. We identified 179,872 tweets, or roughly more than 50% of the tweets in corpus, that discuss RCEP as a domestic issue. Strategic issues were represented by 141,541 tweets, or around 40% of the total tweets about RCEP. In contrast, economic is the key issue in only 23,873 tweets, or about 7% of the total tweets (see Fig. 5). A more detailed reading of the dates provide interesting insights regarding RCEP. At its inception in 2012, there were relatively few discussions about RCEP. This is due to the non-open nature of the RCEP negotiations, meaning there was no intense debate on Twitter. There were only 2080 tweets relating to RCEP in 2014. This increased to 5785 tweets in 2015, again to 23,224 tweets in 2017, then falling to 8097 tweets in 2018. The number surged to 186,712 tweets in the year 2019 before settling on 83,154 tweets in 2020 (see Fig. 6).

This annual variation pattern suggests the overall degree of importance of the RCEP negotiation rounds. In the 2014–2015 period, the outcomes of each round of negotiations were not disclosed in depth by the RCEP negotiators, primarily due to the vast number of sensitive issues. This meant that publicly, there was little to discuss. This is likely the reason behind the low number of tweets about RCEP in 2014 and 2015. However, in 2016, RCEP negotiations intensified, with six rounds of negotiations held every 2 months, with specific issues being discussed ranging from issues of economic and technical cooperation and the chapter on small and medium enterprises (SMEs). This resulted in more discussions about RCEP in public spheres such as Twitter.

**Fig. 7:**
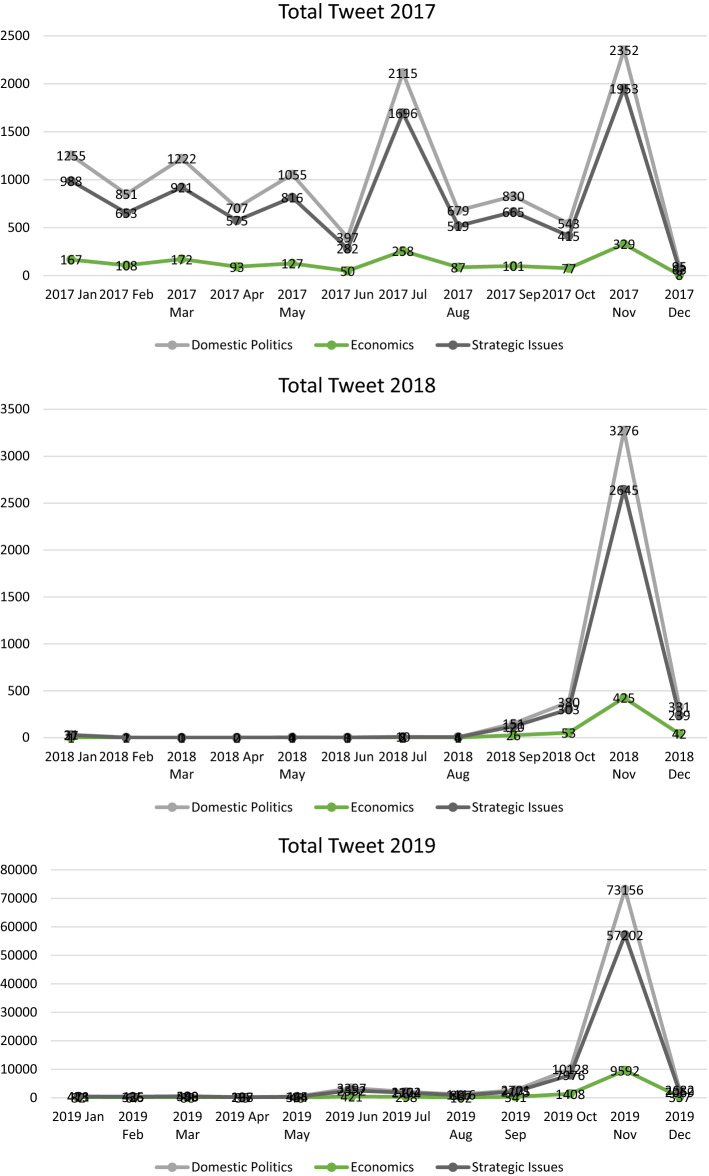
Distribution of RCEP tweets per month (2017–2020)

Since 2017, the RCEP member countries agreed to conduct annual RCEP leaders’ meetings every November to recap the year’s discussions. By examining the time periods, we can see that the RCEP leaders meeting attracted a significantly higher number of tweets than other rounds of negotiations in the same year (Fig. 7). The first RCEP summit was held on 14 November 2017 in Manila, Philippines, attracting 4634 tweets. Three months prior to this summit, there was surge of Twitter discussion during the 19th Round of RCEP Negotiation in Hyderabad, leading to 4069 tweets. These two events represented roughly 30% of the whole tweets on RCEP that year, with the discussion mainly revolving around progress on trade negotiations, and the reaffirmation of leaders’ commitments to negotiations.

In 2017, domestic issues represented the bulk of tweets about RCEP, representing around 50% of relevant tweets that year. Our machine learning model finds that during the first RCEP leaders meeting in 2017, campaigns from several civil societies against RCEP negotiations dominated the Twitter discussion. Meanwhile, debates on strategic issues revolved around how (Trans-Pacific Partnership (TPP) could also balance RCEP and the future of Europe–Asia relations. There was also a discussion about investor to state dispute settlement (ISDS), which would allow investors to sue RCEP governments. There were relatively few Twitter-based conversations about economic issues in 2017, with just 329 tweets being categorised as primarily economic during this meeting. Such tweets tended to discuss who will benefit from ASEAN economic integration.

The second RCEP summit was held in Singapore exactly a year later, on 14 November 2018. This summit focused on the discussion regarding the substantial progress in RCEP negotiations. On Twitter, the summit has attracted 6346 tweets, or 78% of the total tweets in 2018. Several talks in 2018 resulted in breakthrough outcomes, such as the conclusion of two chapters at the 23rd round of negotiations: Customs Procedures and Trade Facilitation (CPTF) and Government Procurement. The CPTF Chapter covers general principles and specific commitments to ensure the transparency and predictability in applying customs laws and regulations of RCEP Participating Countries, while the Government Procurement Chapter aims to promote the transparency of government procurement. Progress was also made regarding market access negotiations and the technical conclusion of the Chapter on Dispute Settlement, as well as various text negotiations on different issues.

The 3rd RCEP summit, which was held in Vietnam in 2019, was the most important RCEP summit since its inception in 2011. The summit was held alongside other summits involving major powers in Asia, including the 16th ASEAN-India summit and the 14th East Asia Summit. This summit led to 139,995 tweets being sent about RCEP, or around 40% of the total tweets in our corpus.

The 3rd RCEP summit was the culmination of 2 years of negotiations during which countries worked to complete RCEP’s core substantial issues, such as market access negotiations. Some of the agenda items decided in the 2019 negotiations were the issue of market access, regulatory texts, and matters related to SMEs, as well as economic and technical cooperation. Market access negotiations include trade in goods, trade in services (financial services and telecommunications), and investment.

What caught the attention of Twitter users in 2019, however, was India’s exit from RCEP negotiations at the November meeting. Although India had been involved in the RCEP negotiation process from the beginning and for all 20 chapters of the agreement, and despite the efforts of other RCEP member countries to make concessions to India, at the 3rd leaders’ meeting, India decided not to sign. This was a surprising move given that a day prior to the leaders’ meeting on 4th November, the discussion surrounding India’s involvement highlighted the win–win outcome for India. At the time, many tweets encouraged India not to join RCEP, primarily analysing domestic issues.

The 4th RCEP summit was held virtually due to COVID-19 in November 2020 and seemed to be an anti-climax for RCEP negotiations. At this summit, 15 leaders finally signed the RCEP agreement. This ceremonial summit attracted 50,360 tweets, representing 60% of the total RCEP tweets during that year. Around 26,270 tweets discussed RCEP as a domestic issue, 20,624 tweets revolved around RCEP as a strategic issue, and the other 3466 tweets discussed RCEP as an economic issue. The majority of the negotiation agenda had been finalised in the previous years.

### Sentiment analysis of RCEP discussions

**Fig. 8:**
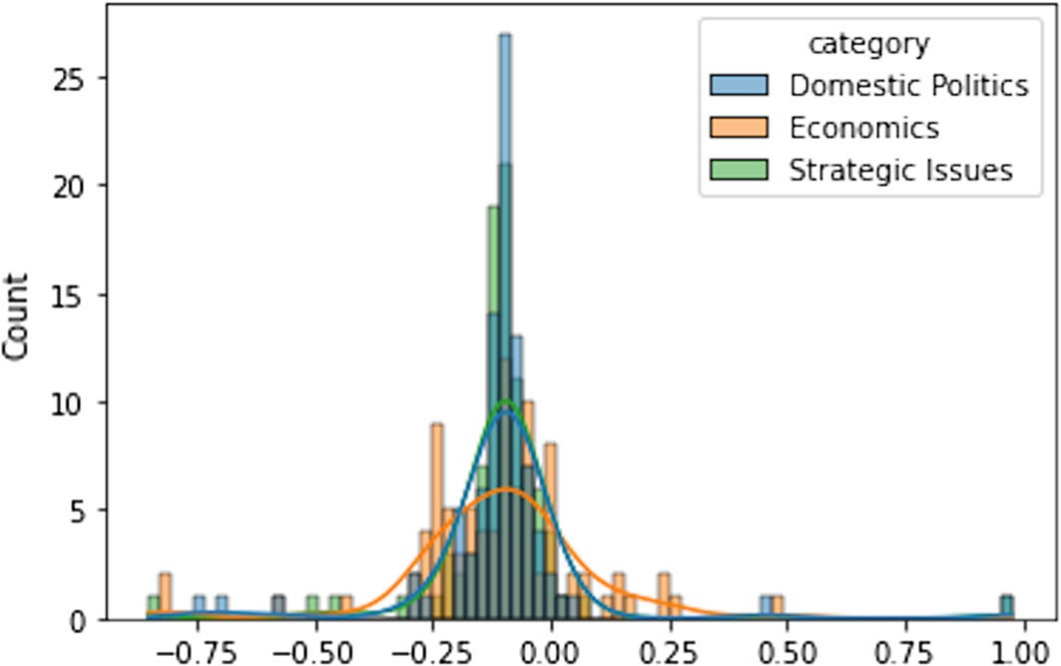
Distribution of average sentiment score for each category

**Fig. 9:**
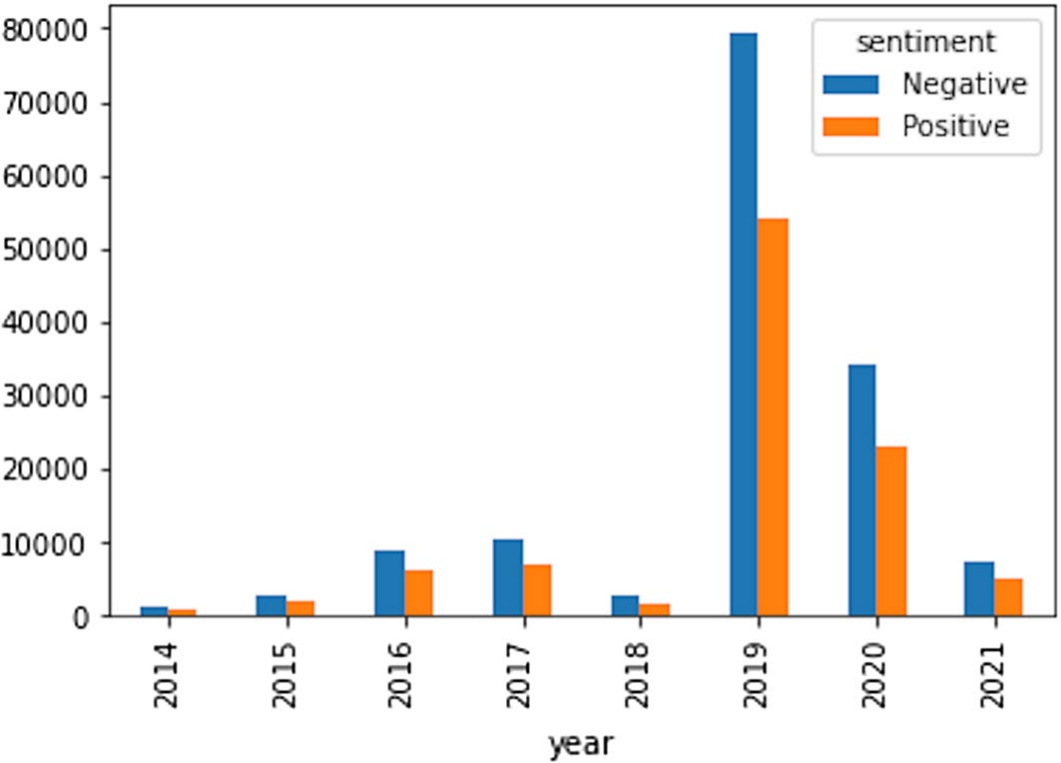
Distribution of positive and negative tweets (annual)

**Fig. 10:**
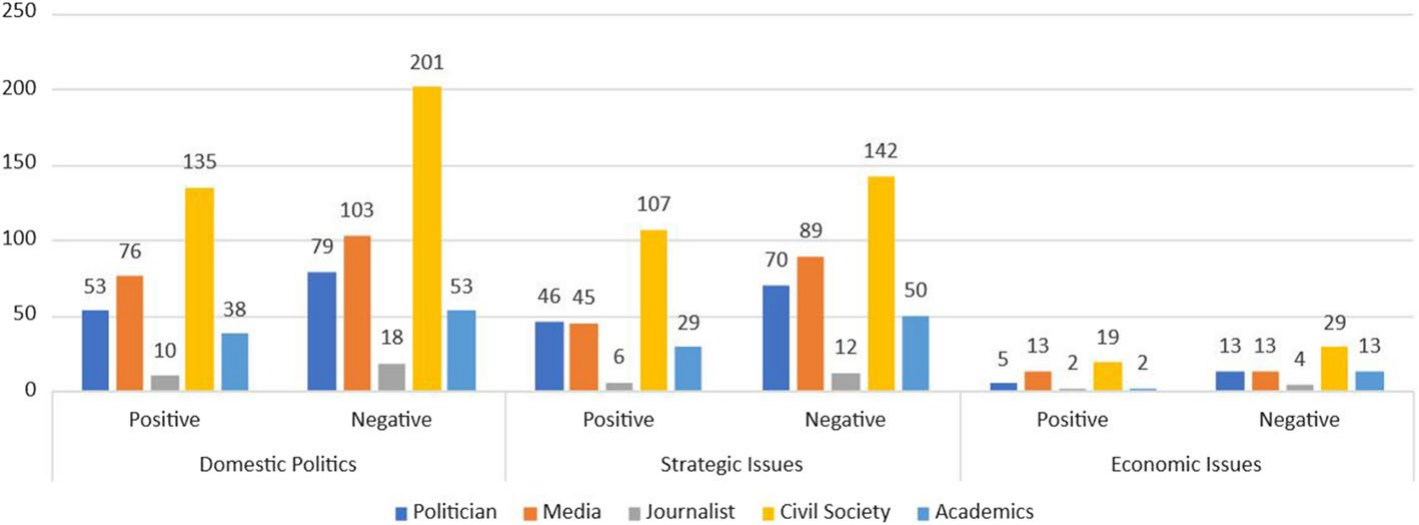
Sentiment analysis of top 20 retweeted accounts

Having unpacked the topic classifications of Twitter discussions about RCEP, we further examine the public sentiment behind each topic. Our findings show that the overall sentiment of Twitter users who discussed RCEP is neutral. The mean score for domestic, economic, and strategic issues is below zero. However, the sentiment is slightly negative when RCEP was framed as a strategic issue (Fig. 8). The table below shows that the sentiment score for tweets about RCEP that were labelled as about strategic issues have a mean of − 0.091, while tweets labelled as about domestic and economic issues have mean scores of − 0.08993 and − 0.08735 respectively although statistically it is not significant (see Table 2). This indicates that, on average, in 2017–2020, public sentiment towards RCEP as a strategic issue is slightly more negative than RCEP as a domestic issue (see Fig. 9). However, in absolute terms, discussion about RCEP’s domestic issues attracted negative tweets the most, with 106,924 negative tweets (59.7% of all tweets on domestic issues) compared with 84,359 negative tweets on strategic issues (59.6% of all tweets on strategic issues) and 14,261 negative tweets on economic issues (58.84% of all tweets on economic issues) (see Fig. 10).

**Table 2:** Overall sentiment score #RCEP

	RCEP	Domestic	Economic	Strategic
Mean	− 0.090389	− 0.089938	− 0.087358	− 0.091465
Std	0.786080	0.786376	0.786858	0.785581
Min	− 0.88856	− 0.882856	− 0.881624	− 0.881624
Max	0.98898	0.982898	0.98118	0.982585

Despite being developed by ASEAN as part an effort to increase its centrality, most of the negative strategic discussions regarding RCEP were about how RCEP could be seen as an extension of China’s Belt and Road Initiative (BRI). After of the signing of RCEP, much of the negative discussion focused more on the strategic impact of RCEP toward other countries. For instance, discussions from outside RCEP countries focused on how RCEP is seen as an initiative that reminds the Western bloc of the importance of joining forces to protect Western power. This can be seen in the tweets by Manfred Weber (@ManfredWeber), a German politician who has served as Leader of the European People’s Party in the European Parliament since 2014, who often tweeted about the importance of a common strategy for the US and Europe. Weber argued that RCEP should be a catalyst for the restart of the US–EU Partnership, given China is not a friend of the European way of life. Other discussions by other users focused on how India’s involvement in RCEP would only increase dependence on China and hence also increase India’s vulnerability.

Negative discussions about the domestic aspects of RCEP was largely dominated by domestic actors’ resistance toward market liberalisation. Meanwhile, domestic discussions labelled as having positive sentiment tended to support the framing of the government in securing domestic interests. During the 3rd RCEP meeting, Indian government officials, notably Piyush Goyal, one of Modi’s high-ranked cabinet minister, reassured Indian citizens that the government would protect the Indian interest. Some domestic actors, such the High-Level Advisory Group (HLAG) headed by economist Surjit S. Bhalla, also discussed the domestic aspects of RCEP that our model labelled as positive.

Negative discussions in India on economic issues relating to RCEP revolved around the massive economic slowdown faced by China and how RCEP might reduce the program. Other discussions focused more on the fear that decreasing tariffs for Beijing would flood local Indian markets with cheap Chinese goods, throttling local industries and widening the country’s trade deficit. Positive sentiment over economic issues, on the other hand, discussed how there might be mutually beneficial outcomes from RCEP. Tweets that discussed the possibility of India’s signing of RCEP were also labelled as positive. In general, across the top twenty most retweeted domestic actors were mostly view RCPE as negative as strategic, economic, and domestic issues (see Fig. 10).

**Fig. 11:**
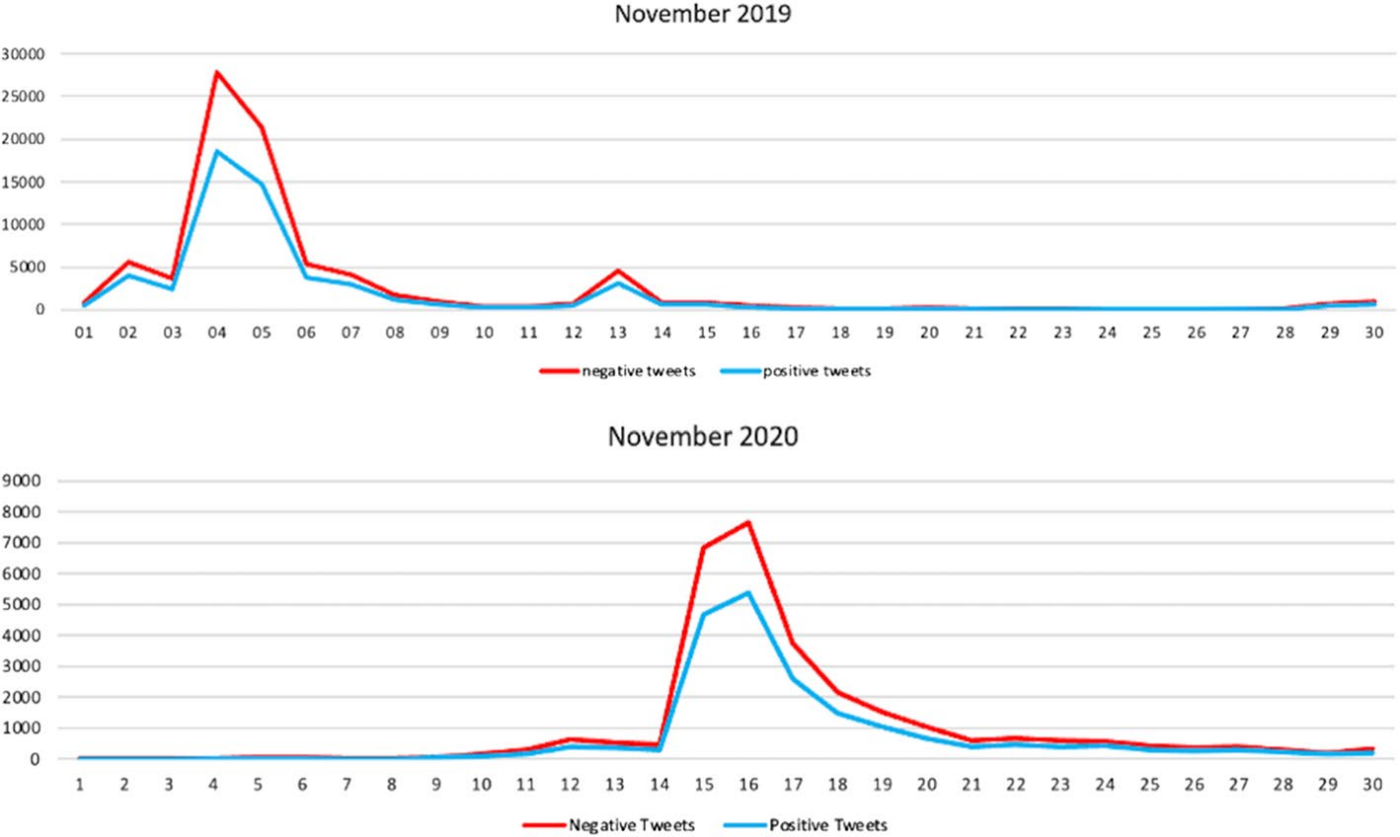
Distribution of positive and negative tweets in November 2019 and November 2020

**Fig. 12:**
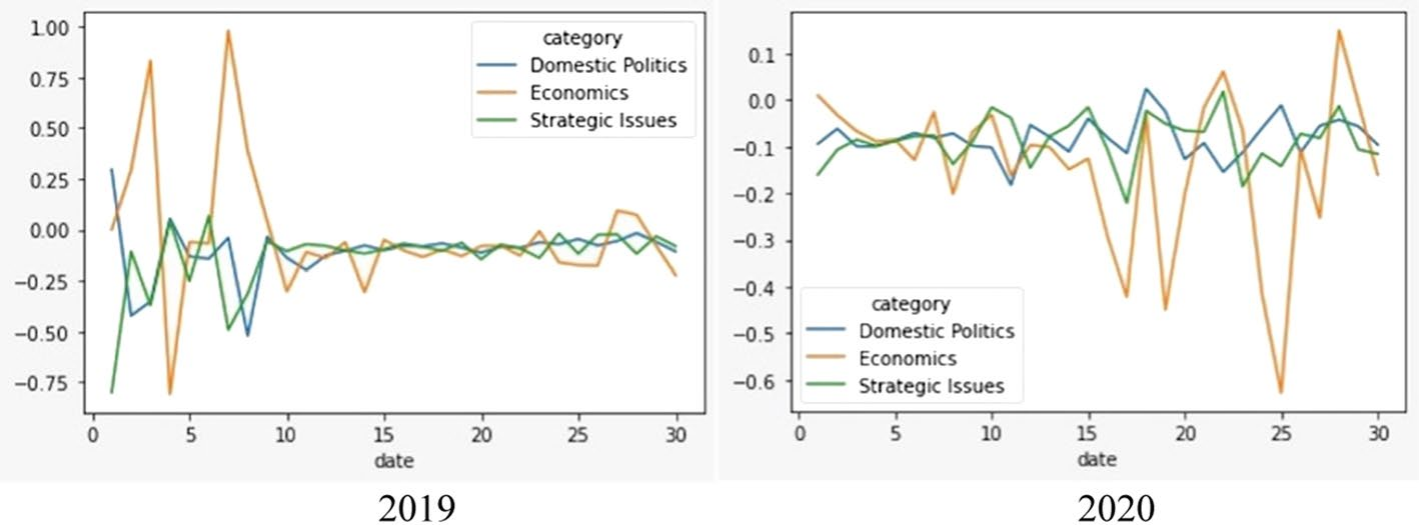
Sentiment score in RCEP Leaders’ meeting 2019 and 2020

Given that the largest proportion of tweets from our corpus come from 2019 and 2020, we drill down the data into November 2019 and November 2020. Using line plots and histograms, we see the average daily trend of sentiment analysis scores for each category. By mapping these categories and the number of tweets into a histogram, we can explore the distribution of sentiment analysis scores. Our data shows that the most intense RCEP discussions occurred during two events: the 2nd RCEP leaders’ meeting on 4 November 2019 and the 3rd RCEP leaders’ meeting on 15 November 2020. The graph in Fig. 11 shows that tweets spike dramatically around the time of these two events, while Fig. 12 displays the difference in sentiment scores across each category. At the beginning of the meeting, the sentiment score for discussions about economic issues was positive, but on the day of the leaders’ meeting itself, sentiment scores from discussions of economic issues decreased and became negative, before returning to positive several days later. The same situation occurred with the sentiment scores for discussions on the domestic and strategic issues relating to RCEP. Although there is slightly different degree of public sentiment regarding domestic and strategic issues, overall, the sentiment is below zero, indicating a negative sentiment.

The RCEP leaders meeting in 2020 was an important moment in RCEP negotiations. This meeting became the key venue for negotiating member countries to agree on final form of the agreement. Unlike in 2019, the 2020 sentiment score was positive for domestic and strategic issues, but the overall sentiment score on economic issues was negative. Debates relating to economic issues were also much more volatile, with the sentiment moving to negative, touching positive, then plunging into negative again in the days after the leaders’ meeting. This is because there were large discussions on Twitter exploring the negative impact of RCEP each member country’s economy.

### Network analysis of RCEP discussion

Having discussed the overall sentiment of public discussion about RCEP, the next question is which actors dominated the conversation. To understand this, we first generate data about Twitter accounts that engaged with RCEP. There were three types of users: first, users who were primarily active in tweeting about RCEP. Second, users who primarily got retweeted. Third, users who were most likely to get a reply. The most active users can inform us about which actors pay more attention to RCEP negotiations—active tweeting about RCEP means they are directly interested. The most retweeted accounts might provide insights on how to understand the flow of political information. Meanwhile, the accounts which receive the most replies can show which actors generate dynamic conversations. As suggested by other scholars [56], a Twitter reply can be seen as a site for politically engaged public conversation.

**Fig. 13:**
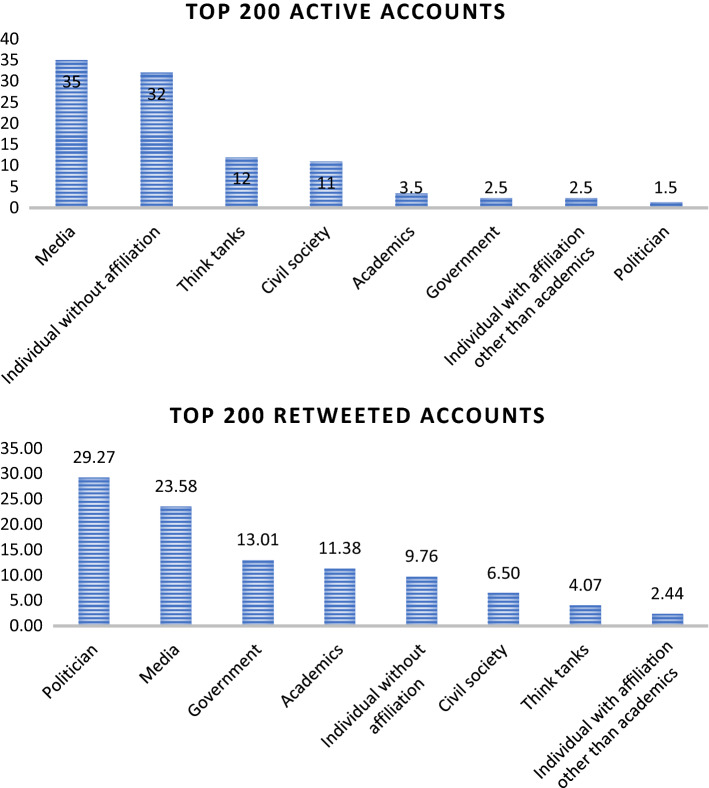
Top 200 active accounts and retweeted accounts

At the second stage, we label the top 200 hundred users of each of the above types into eight broad categories: politician, media, think tank, academics, civil society, government, individual with affiliation, and individual without affiliation (ordinary citizens). We find that the media are the users who most frequently discuss RCEP, followed by ordinary citizens, civil societies, academics, and government accounts. However, when we look at who receives the most retweets, politicians come first, followed by media, government, and academics. This shows that politicians have more reach in RCEP discussions than any other actors. Interestingly, even though politicians, government agencies, and academics are a lot less active in discussing RCEP, their tweets are more likely to be retweeted. Thus we can argue that the public sees these actors as a key source of information (see Fig. 13).

Furthermore, our data regarding top replies also provides interesting insights. We drill down to find the topmost replied accounts that engaged on domestic, economic, and strategic issues relating to RCEP. We find that ordinary citizens and media dominated all three issues. Around 45% of the total 200 accounts most replied to on domestic issues are ordinary citizens. However ordinary citizen only reply for around 28% in strategic issues and 24% in economic issues. Actors categorized as media represent 19% of the most active users who responded to RCEP conversations as domestic issues, 27% in RCEP as strategic issues, and 22% in RCEP as economic issues. This shows that the ordinary citizen as a category of users gets more replies on domestic issues, followed by strategic issues and economic issues. Unlike ordinary citizens, the media primarily handles RCEP as a strategic issue, followed by economic issues and then domestic issues.

**Fig. 14:**
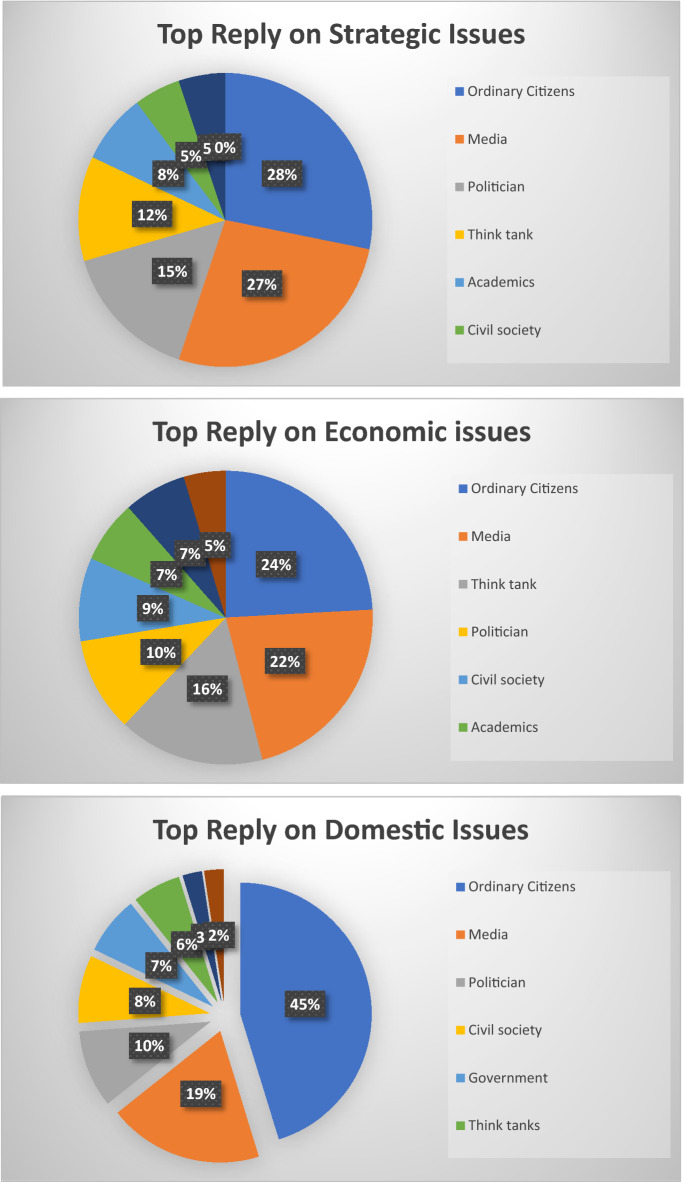
Top 200 replies on strategic, economic, and domestic issues

However, beyond ordinary citizens and media, there are significant differences in how the public discusses RCEP on Twitter. In the context of conversations about RCEP as a domestic issue, politicians (19%) and civil society (10%) are the two categories of actors who get the most replies. Meanwhile, in conversations related to strategic issues, politicians (15%) and thinktanks (12%) received the most replies. Finally, in conversations related to economic issues, think tanks are at the top with 22%, followed by politicians at 10% (Fig. 14).

We now further look at the network analysis of Twitter discussions regarding RCEP. From the result of text categorisation and sentiment analysis, the dataset was transformed into a form that included the source, the target, and the number of replies. Figure 14 illustrates the positioning of the most influential actors in the RCEP network and subnetworks. Each grey node represents a user involved in the discussion. The connecting lines symbolise the relationship formed by tweet replies between users, and each circled node represents clusters that are centered on an influential actor. The top five Twitter users who received the most replies on RCEP were: @narendramodi (politician representing government), @PiyushGoyal (politician representing government, @RahulGandhi (opposition politician), @ShekharGupta (journalist), and @GlobalTimesNews (media). The fact that @GlobalTimesNews manages to be one of the top five users with the largest network is particularly interesting. While the other four users are based in India, from where the majority of our corpus originates, The Global Times is a Chinese-sponsored media outlet that engages with international audience. This indicates that Chinese-sponsored media influenced discussions on RCEP in the Twittersphere, despite Twitter being officially blocked in China.

Moreover, when examining the clusters that appear, clusters around @narendramodi and @GlobalTimesNews are the most populated and networked. Table 3 lists the centralities of Twitter usernames in RCEP networks as the output of network measurements using the Python package NetworkX. In general, @narendramodi is recognised as the most influential actor in RCEP networks, as he gains the most value at almost all centralities. Since the degree centrality of @GlobalTimesNews and @narendramodi are approximately equal in every network, it indicates a similar persona among those accounts, despite their very different identities (Indian politician versus Chinese state-supported media outlet). Surprisingly, the outstanding eigenvector centrality of @narendramodi proved that the surrounding users of @narendramodi are much more important and popular compared to the surrounding of the other users. @narendramodi’s eigenvector six times surpasses the eigenvector of other public figures and reputable organizations such as @PiyushGoyal, @RahulGandhi, @ShekharGupta, and @GlobalTimesNews. Compare with @narendramodi, @GlobalTimesNews has a very low eigenvector centrality meaning that its surrounded by unimportant users. However, betweenness centrality in general and domestic issues shows @GlobalTimesNews outperformed @narendramodi in bridging users to interact with each other.

**Table 3:** Centrality in #RCEP

Topic	Degree centrality		Eigenvector centrality		Betweenness centrality	
	Username	Value	Username	Value	Username	Value
General	**narendramodi**	**0.025821949**	**narendramodi**	**0.674558815**	**globaltimesnews**	**0.064704048**
	globaltimesnews	0.021017865	PiyushGoyal	0.146411915	narendramodi	0.047270716
	PiyushGoyal	0.018165441	RahulGandhi	0.084405875	vish2bnice	0.044484226
	RahulGandhi	0.017940249	PMOIndia	0.074321638	PiyushGoyal	0.040495537
	ShekharGupta	0.015463144	jotendra_kumar	0.052894966	RahulGandhi	0.028717176
Domestic politics issues	**narendramodi**	**0.021568627**	**narendramodi**	**0.687494814**	**globaltimesnews**	**0.069541551**
	globaltimesnews	0.020299885	globaltimesnews	0.10745932	vish2bnice	0.043893467
	RahulGandhi	0.015109573	jotendra_kumar	0.063997813	narendramodi	0.031768894
	PiyushGoyal	0.014417532	PiyushGoyal	0.062452418	PiyushGoyal	0.027084978
	ShekharGupta	0.012802768	RahulGandhi	0.062384302	ShekharGupta	0.025491687
Economic issues	**narendramodi**	**0.016995614**	**narendramodi**	**0.695879678**	**narendramodi**	**0.002712056**
	globaltimesnews	0.01370614	MayankSaraf51	0.144745887	PMOIndia	0.002213433
	RahulGandhi	0.010964912	ykamath	0.130512905	ykamath	0.001871193
	PiyushGoyal	0.010964912	AjayKushwaha_	0.128460638	heggere_nawaz	0.001793001
	ShekharGupta	0.00877193	IronManIndianDr	0.128460638	globaltimesnews	0.001539178
Strategic issues	**narendramodi**	**0.022156573**	**narendramodi**	**0.695720228**	**narendramodi**	**0.045763081**
	globaltimesnews	0.018530952	PiyushGoyal	0.087401078	globaltimesnews	0.039944377
	PiyushGoyal	0.015173896	progressbharat1	0.066702456	PiyushGoyal	0.033487067
	RahulGandhi	0.012756815	SatisValaganth	0.066441199	vish2bnice	0.033475998
	ShekharGupta	0.012219686	PMOIndia	0.066230226	EvanFeigenbaum	0.024606677

In this table, the bolded clusters represent the most populated and networked clusters within our analysis, indicating a higher degree of interaction and engagement among users discussing the respective topics

**Fig. 15:**
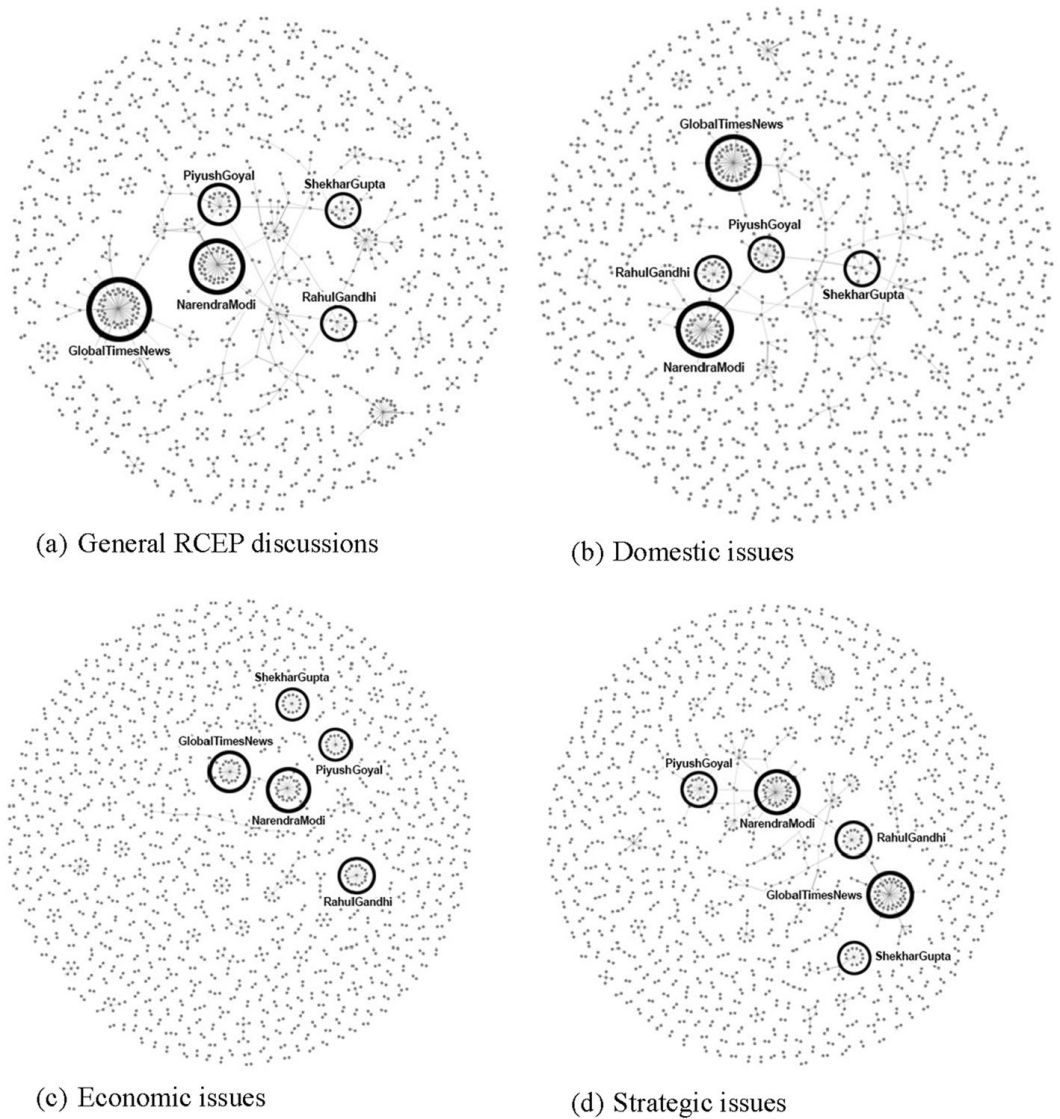
Network analysis of RCEP discussions

Figure 15 shows how the top five users have different interactions with other users depending on whether they discuss RCEP as a domestic, economic, or strategi issue. In discussions about domestic and strategic issues, both Narendra Modi as a politician representing government and Rahul Gandhi as an opposition politician have more interaction in domestic and strategic discussions of RCEP than economic discussions. This might indicate that discussing RCEP as a domestic and/or strategic issue may attract users with oppositional stances to interact with one another. While in the economic issue, there is hardly any interaction between Narendra Modi and Rahul Gandhi, indicating that discussing RCEP as an economic economic issue does not attract any interaction between the two oppositional stance to interact.

## Conclusion

This article has shown the benefit of using the big data analytics approach in unpacking discussions about RCEP on Twitter. This approach provides us with several insights. First, our research contributes to the debate regarding how the public contextualises ongoing free trade negotiations as primarily an issue relating to domestic, economic, and/or strategic concerns. Our research shows that domestic issues dominated RCEP discussions on Twitter, alongside strategic issues. In contrast, RCEP as an economic issue is seemingly under-discussed relative to the other two issues. This shows that despite its direct focus on economic issues, RCEP did not attract significant public discussion from an economic angle. Second, our research finds that the overall public sentiment toward RCEP is negative. These negative sentiments are mainly driven by fear of China’s geopolitical ambitions, domestic protectionist agendas, and the negative impact of RCEP on domestic economies. However, surprisingly, contextualising RCEP as a strategic issue has a more negative sentiment compared to discussing RCEP as domestic and economic issue. Third, our network analysis suggests that different discussions regarding RCEP may attract different types of actors. This article also finds that conversations that place trade negotiation as domestic issues are primarily influenced by politicians and civil society; debates on trade negotiations as strategic issues are influenced by politicians and thinktanks; and free trade discussions about economic issues are influenced by thinktanks and politicians.

These findings contribute to our understanding of domestic actors engagement in public conversations about free trade as an economic, domestic, and/or strategic issue by providing insights on how Twitter users contextualise their opinions. Our research enables to further unpack how the public sees free trade in a variety of contexts, with our findings showing that free trade is contextualised much more frequently as issues relating to domestic and strategic concerns rather than ones of economic issues. This shows that policymakers should pay more attention not only the importance of economic gain from free trade, but also the domestic and strategic impacts of such endeavours. In addition to substantive debate, we also contribute to the growing use of big data analytics approaches to understand international negotiations. The combination of topic classification, sentiment analysis, and network allow us to explore the sentiment as well as identify influential actors on each topic of RCEP discussion. Most studies applying big data analytics approaches tend to focus on events happening in the Global North, such as the Brexit negotiation and TTIP. Our research brings Asia into the literature by examining the case of RCEP.

Although our research identifies the influential actors in RCEP discussions, we did not explore the left–right divide in these discussions as suggested by Milner and Judkins [34]. Instead, we focused on identifying the different domestic groups, such as politicians, media, and civil society. Future research could delve deeper into the political ideologies and positions held by different actors on RCEP, and explore whether there is indeed a left–right divide on this issue. This would provide a more nuanced understanding of the RCEP discourse and the factors that shape public opinion on free trade agreements.

The application of big data analytics is not without limitation. Our use of English language has made our analysis is highly skewed toward countries which primarily use English as their main language, such as India, which dominates our corpus. Our findings are thus heavily influenced by how India’s Twittersphere discusses RCEP. Considering India’s tendency to be more protectionist, negative sentiment towards RCEP in general reflects Indian public sentiment towards international trade agreements that may not benefit India. In addition, the lack of conversation about the economy and the dominance of domestic and strategic issues in RCEP discussions also reflects India’s view of RCEP as a strategic and domestic threat to India. As a result, this research cannot elaborate about RCEP discussions and public sentiment in other countries such as China, South Korea, Japan, and Indonesia given the language differences. Future research should be able to provide a comparative analysis between countries participating in RCEP, and it would be useful to compare what topics are discussed in different countries as well as any difference in sentiment analysis.

## Data Availability

The data that support the findings of this study are available from the corresponding author, MF Karim, upon reasonable request. The data can be obtained in this link Data for reviewer.
